# Retrospective study of predictive factors for postoperative complications of hepatectomies lasting 12 or more hours

**DOI:** 10.20407/fmj.2019-003

**Published:** 2020-02-11

**Authors:** Mariko Nagata, Yoshitaka Hara, Seiko Hayakawa, Hidefumi Komura, Junpei Shibata, Chizuru Yamashita, Tomoyuki Nakamura, Naohide Kuriyama, Sohta Uchiyama, Kotaro Kawata, Osamu Nishida

**Affiliations:** 1 Department of Anesthesiology and Critical Care Medicine, Fujita Health University, School of Medicine, Toyoake, Aichi, Japan; 2 Department of Internal Medicine, Mitoyo General Hospital, Kanonji, Kagawa, Japan

**Keywords:** Extremely long hepatectomy, Postoperative complications, Predictive factors

## Abstract

**Objectives:**

Hepatectomy is used to treat several liver diseases, although perioperative mortality and postoperative complication rates remain high. Given the lack of relevant studies to date, the present study aimed to investigate potential predictive factors for postoperative complications in patients undergoing hepatectomies lasting 12 or more hours (termed “extremely long hepatectomies”).

**Methods:**

Adult patients undergoing treatment in the intensive care unit (ICU) after extremely long hepatectomies at Fujita Health University Hospital between 2014 and 2017 were enrolled in the study. Postoperative complications were classified as “major complications” and “non-major complications” according to the Clavien–Dindo Classification grading system. We also divided our study population into “simple hepatectomy” and “non-simple hepatectomy” subgroups for further analysis. Statistical analyses were performed using the Mann–Whitney U test, chi-squared test, and multiple logistic regression analysis.

**Results:**

In total, 114 patients (Major Complications Group, n=44; Non-Major Complications Group, n=70) were enrolled. In the Simple Hepatectomy Group, there were no significant variables. In the Non-Simple Hepatectomy Group, female sex (odds ratio [OR], 13.4; 95% confidence interval [CI], 1.00–1.81×10^2^; *p*=0.04) and lactate levels at ICU admission (OR, 1.6; 95% CI, 0.99–2.59; *p*=0.05) were independent factors associated with major postoperative complications.

**Conclusions:**

In the Simple Hepatectomy Group, there were no significant variables. In the Non-Simple Hepatectomy Group, female sex and lactate levels at ICU admission of patients who underwent extremely long hepatectomies may be independent factors associated with major postoperative complications.

## Introduction

In recent years, hepatectomy has been used as a treatment for multiple liver diseases. Although preoperative management and surgical techniques have improved over the years, the perioperative mortality rate (0.24%–9.7%) and postoperative complication rate (4.09%–47.7%) remain high.^1^ Previous reports have shown that elevated lactate levels,^2^ reduced muscle mass,^3^ and surgical duration^4^ are predictive factors for postoperative complications following relatively short-duration hepatectomies. However, no studies have been performed regarding predictive factors for postoperative complications of hepatectomies lasting 12 or more hours (termed “extremely long hepatectomies”). The results of a previous study conducted by our group indicated that lactate levels at intensive care unit (ICU) admission may be a predictive factor for posthepatectomy liver failure following extremely long hepatectomies.^5^ However, in that study we conducted only a univariate analysis of this issue. Other serious postoperative complications apart from posthepatectomy liver failure, such as postoperative infections, hemorrhage, and bile leakage, also occur following hepatectomy.^1^ Thus, there is great significance in investigating anew the predictive factors for postoperative complications. Here, we report on our exploratory investigation of predictive factors for postoperative complications of patients undergoing ICU care following extremely long hepatectomies.

## Methods

### Selection criteria for study participants and baseline characteristics

Consecutive patients aged 18 years and older undergoing ICU care following extremely long hepatectomies, performed at Fujita Health University Hospital between January 2014 and December 2017, were enrolled in the present study. Patients who had undergone liver transplantations were excluded. Demographics comprising age (years), sex, height (cm), and weight (kg), and clinical information including body mass index (kg/m^2^), history of diabetes mellitus (DM) (yes/no), preoperative cholangitis (yes/no), cirrhosis (yes/no), American Society of Anesthesiologists Physical Status (ASA PS), Child–Pugh score, adjusted ICGR_15_ (indocyanine green retention rate at 15 min) (%), surgical procedure (simple hepatectomy or non-simple hepatectomy), major hepatectomy of two or more Couinaud segments (yes/no), surgical approach (open hepatectomy or laparoscopy), redo hepatectomy (yes/no), surgical duration (h), blood loss (mL/kg/h) during surgery, infusion and blood transfusion volume (per unit time) during surgery, intraoperative use of noradrenaline (yes/no), blood inflow occlusion (yes/no), lactate levels at ICU admission (mmol/L), liver failure (n), 28-day survival (%), 90-day survival (%), and length of hospital stay (days) were collected for each study participant. These data were retrieved from the patients’ electronic medical records and the ICU database.

### Anesthesia management

Anesthesiologists were responsible for anesthesia management during each patient’s surgery. Ringer’s acetate, Ringer’s bicarbonate, and colloid solutions were used for the intraoperative infusion. Ringer’s lactate solution was administered to some patients at the time of admission to the operating room and continued at the time of induction of anesthesia; however, these patients were not excluded from the study because the concentration of Ringer’s lactate solution used was very low.

### Definitions

Postoperative complications were defined as complications that occurred following surgery until the patient was discharged from the hospital. These were retrieved from the patients’ electronic medical records and graded according to the Clavien–Dindo (C-D) classification.^6^ “Major Complications” are defined as Grade IIIa complications and higher according to the C-D Classification, and identifies patients requiring invasive therapy. Complications categorized as below Grade IIIa and no postoperative complications were defined as “Non-Major Complications.” The details and numbers of the postoperative complications that occurred in each C-D classification group of patients are presented in Supplementary Table 1.

In this study, posthepatectomy liver failure was defined as Grade B or C liver failure according to the International Study Group of Liver Surgery (ISGLS) criteria^7^. Under the ISGLS criteria, Grade A liver failure is defined as liver failure not requiring clinical intervention; hence, Grade A patients were not included in the present analysis. Grade B is deviation from clinical management without invasive treatment, and Grade C is deviation from clinical management requiring invasive treatment.

“Major hepatectomy” was defined as resection of two or more segments of the liver as indicated by the Couinaud classification.

“Simple hepatectomy” was defined as hepatectomy alone or hepatectomy with cholecystectomy. By contrast, “non-simple hepatectomy” was defined as liver resection with extrahepatic procedures such as blood vessel/bile duct resection and reconstruction, or combined resection with other organs (such as digestive tract and pancreas). Surgical procedures (including blood vessel/biliary resection) are listed in Supplementary Table 2.

“Blood inflow occlusion” was defined as any inflow occlusion technique, such as the Pringle maneuver and partial and selective inflow occlusions, performed to reduce bleeding during surgery.

Hospital stay was defined as the total number of in-hospital days consisting of both postoperative days and those for preoperative evaluation and management such as biliary drainage, portal embolization, and blood sugar control in diabetic patients.

### Statistical analysis

All data are shown as median and interquartile range. Patients were assigned to the Major Complications Group and the Non-Major Complications Group. These two groups were compared in terms of categorical variables using the chi-squared test and non-categorical variables using the Mann–Whitney U test. Moreover, factors associated with major postoperative complications were analyzed using multiple logistic regression analysis. All analyses were performed using the statistical software Stat Flex ver4 (Artec, Osaka, Japan).

Multiple logistic regression analysis was exploratively conducted on the study results, where the dependent variable was the presence or absence of major postoperative complications. The independent variables were age (years), sex, body mass index (kg/m^2^), history of DM (yes/no), preoperative cholangitis (yes/no), cirrhosis (yes/no), ASA PS, adjusted ICGR_15_ (%) as an indicator of preoperative liver function, surgical approach (open hepatectomy or laparoscopy), major hepatectomy (yes/no), redo hepatectomy (yes/no), blood inflow occlusion (yes/no), surgical duration (h), blood loss (mL/kg/h) during surgery, infusion and blood transfusion volume (per unit time) during surgery, intraoperative use of noradrenaline (yes/no), and lactate levels at ICU admission (mmol/L). Variables for which multicollinearity was a concern were excluded from the analysis.

It has been reported that non-simple hepatectomy is difficult, requires a long surgical duration, and has a high incidence of major postoperative complications.^8^ Therefore, we further divided the subjects into two subgroups, namely “Simple Hepatectomy” and “Non-Simple Hepatectomy,” and explored additional factors contributing to the major postoperative complications for each subgroup.

### Ethical considerations

This study was conducted after receiving the approval of the Institutional Review Board of Fujita Health University (HM19-011). A waiver of consent was obtained because this was a retrospective study.

## Results

### All enrolled patients

Patient baseline characteristics are shown in Table 1. In total, 114 patients were included and were divided into a Major Complications Group (n=44) and a Non-Major Complications Group (n=70). Statistical analysis of the preoperative patient baseline characteristics indicated that there was a significantly higher number of women in the Major Complications Group (*p*=0.041). The Child–Pugh score and ICGR_15_ test score were significantly higher in the Major Complications Group (*p*=0.005 and *p*=0.005, respectively). The rates of non-simple hepatectomy, major hepatectomy, and open hepatectomy were all significantly higher in the Major Complications Group compared with the Non-Major Complications Group (*p*<0.001, *p*=0.002, and *p*<0.001, respectively). Surgical duration was also significantly longer in the Major Complications Group (*p*=0.001). Furthermore, median blood loss per unit time (*p*<0.001), median infusion and blood transfusion volume per unit time (*p*=0.008), and the need for blood inflow occlusion (*p*=0.001) were all significantly higher in the Major Complications Group than in the Non-Major Complications Group. Lactate levels at ICU admission were significantly higher (*p*<0.001) and the length of hospital stay was longer in the Major Complications Group than in the Non-Major Complications Group (*p*<0.001). The 28-day and 90-day survival rates were both lower in the Major Complications Group (*p*=0.007 and *p*<0.001, respectively). Posthepatectomy liver failure was the most common complication and occurred in 44 of the 114 subjects (38.6%). Of the 44 subjects in the Major Complication Group (C-D III and higher), 32 (72.7%) suffered postoperative liver failure.

Multiple logistic regression analysis identified ASA PS Class 3 (odds ratio [OR], 11.5; 95% confidence interval [CI], 0.99–1.34×10^2^; *p*=0.05) and surgical duration (OR, 1.21; 95% CI, 1.01–1.46; *p*=0.04) as independent factors associated with major postoperative complications. In addition, results of exploratory multivariate analysis for female sex (OR, 3.25; 95% CI, 0.91–11.6; *p*=0.07) and open hepatectomy (OR, 4.55; 95% CI, 0.86–24.1; *p*=0.08) approached significance (Table 2).

### Simple hepatectomy and non-simple hepatectomy

In the Simple Hepatectomy Group, the number of open hepatectomies was higher and the hospital stay was longer in the Major Complications Group (*p*=0.038 and *p*=0.001, respectively) than in the Non-Major Complications Group (Table 3). Multiple logistic regression analysis identified no independent factor associated with major postoperative complications in the Simple Hepatectomy Group. However, exploratory multivariate analysis showed a significant trend for female sex (OR, 4680; 95% CI, 0.37–6.0×10^7^; *p*=0.08), history of DM (OR, 1530; 95% CI, 0.41–5.69×10^6^; *p*=0.08), open hepatectomy (OR, 28,500; 95% CI, 0.36–2.24×10^9^; *p*=0.07), blood inflow occlusion (OR, 56.1; 95% CI, 0.16–1.93×10^4^; *p*=0.18), and surgical duration (OR, 2.45; 95% CI, 0.71–8.40; *p*=0.16) (Table 4).

**Figure 1:**
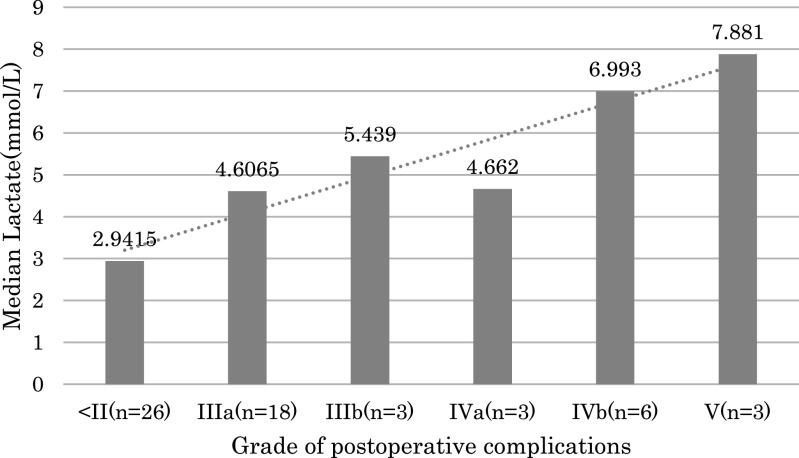
Relationship between Clavien–Dindo (C-D) classification and lactate levels at intensive care unit (ICU) admission in non-simple hepatectomies

The baseline characteristics of patients undergoing non-simple hepatectomies are shown in Table 5. Surgical duration was longer (*p*=0.036) and the rate of major hepatectomy and median blood loss per unit time higher in the Major Complications Group (*p*=0.05 and *p*=0.013, respectively) compared with the Non-Major Complications Group. Blood inflow occlusion (*p*=0.023) and lactate levels at ICU admission (*p*=0.002) were higher in the Major Complications Group. The 90-day survival rates were significantly lower (*p*=0.015) and hospital stay significantly longer (*p*<0.001) in the Major Complications Group than in the Non-Major Complications Group. Multiple logistic regression analysis identified female sex (OR, 13.4; 95% CI, 1.00–1.81×10^2^; *p*=0.04) and lactate levels at ICU admission (OR, 1.6; 95% CI, 0.99–2.59; *p*=0.05) as independent factors associated with major postoperative complications in the Non-Simple Hepatectomy Group. In addition, exploratory multivariate analysis showed a significant trend for ASA PS Class 3 (OR, 80.8; 95% CI, 0.80–8.08×10^3^; *p*=0.06), surgical duration (OR, 1.30; 95% CI, 0.94–1.80; *p*=0.10), and infusion and blood transfusion volume (OR, 1.46; 95% CI, 0.88–2.42; *p*=0.13) (Table 6). Based on these results, we wanted to determine whether an increase in lactate levels at ICU admission could be associated with the risk of major postoperative complications, and to clarify whether the severity of C-D grade was related to an increase in lactate levels. The median lactate levels at ICU admission by C-D grade are shown in Figure 1. An increase in C-D grade correlated with an almost correspondent increase in lactate levels; the median lactate level at C-D Grade IIIa was 4.6065 mmol/L.

## Discussion

Hepatectomies are still associated with a large number of complications and a high mortality rate, indicating that preoperative assessment, the surgical procedure employed, and the perioperative care patients receive are of critical importance. Previous studies have investigated predictive factors for posthepatectomy complications but have focused on surgeries of short duration. In the present study, we investigated the predictive factors for major postoperative complications following extremely long hepatectomies.

The results indicated that, in enrolled patients, ASA PS Class 3 and surgical duration were independent factors associated with major postoperative complications. In addition, this study was limited to exploratory studies because the number of cases was not sufficient. For all enrolled patients, the results suggested that female sex and open hepatectomy may be independent predictive factors of major postoperative complications. ASA PS is a method defined by the American Society of Anesthesiologists to evaluate the general condition of preoperative patients in 6 stages, whereby Class 3 or higher is considered high risk.^9^ In addition, prognosis can be correlated with ASA PS.^10^ In this study, for patients undergoing extremely long hepatectomies, ASA PS Class 3 was an independent factor in predicting the risk of major postoperative complications. This result indicates that the preoperative evaluation should be strictly carried out and that patients falling under ASA PS Class 3 should be identified as being at high risk of major postoperative complications. Major postoperative complications may be reduced in these patients by stabilizing their general condition prior to surgery.

Previous reports have shown that surgical duration^4,11^ was a predictive factor for major postoperative complications. Similarly, the results of this study suggest that surgical duration may also be a predictive factor with regard to extremely long hepatectomies.

The reason for the lengthy surgical duration may be that, in this study, there were many cases of hepatectomies performed with blood vessel or bile duct resection and reconstruction, or cases whereby combined resection of the liver with other organs, such as hepatopancreatoduodenectomy, were performed. It has been reported that non-simple hepatectomy is difficult, requires a long surgical duration, and has a high incidence of major postoperative complications.^8^ Therefore, we further divided the subjects into two subgroups, the Simple-Hepatectomy and Non-Simple Hepatectomy groups, and conducted additional multivariate regression analyses. In the Simple Hepatectomy Group, about 20% of patients developed major postoperative complications, but there were no independent factors contributing to major postoperative complications. However, the results of exploratory multivariate analysis in this study suggested that in simple hepatectomy, female sex, history of DM, open hepatectomy, blood inflow occlusion, and surgical duration are possible independent factors in predicting major postoperative complications. In contrast, for the Non-Simple Hepatectomy Group, the results indicated that elevated lactate level at ICU admission was an independent factor predicting postoperative complication incidence. Serum lactate levels have long been targeted for patient monitoring and management in the field of intensive care medicine. Septic shock, which includes in its diagnostic criteria serum lactate levels of 2 mmol/L and above, is a pathologic condition characterized by cellular and metabolic dysfunction associated with circulatory failure that leads to high mortality rates.^12,13^ Thus, in the present study the identification of lactate levels as an independent risk factor that can potentially predict major postoperative complications is of great clinical interest.

In the present study, because the measurement of lactic acid level at ICU admission was measured within approximately 1 hour after surgery, the lactic acid level at ICU admission was expected to be similar to postoperative lactic acid levels. The cause of the increase in lactic acid levels in this study is unknown, although previous reports suggest that elevated lactate levels following hepatectomy could be due to the balance of intraoperative tissue oxygen metabolism,^14^ the effect of ischemia-reperfusion injury to the liver caused by use of the Pringle maneuver,^15^ and reduced lactic acid clearance caused by resection of the liver.^16^ Our results suggest that there may be a need to assess lactate levels during surgery and take appropriate measures to prevent their increase postoperatively.

In the present study, in the Non-Simple Hepatectomy Group we found that the median lactate level at ICU admission was 2.9415 mmol/L for the Non-Major Complications Group and 4.6065 mmol/L for C-D Grade IIIa patients. A higher C-D grade was associated with an increase in lactate levels at ICU admission (Figure 1).

Vibert et al. reported that when lactate levels following hepatectomy exceeded 2.8 mmol/L, severe morbidity of C-D Grade III or higher increased and that when it exceeded 3 mmol/L, the 90-day survival rate was affected.^17^ However, approximately half of their study subjects underwent surgery for less than 5 hours. In addition, their research was mostly on simple hepatectomies. By contrast, the present study showed that the median lactate level of patients with a C-D Grade IIIa in the Non-Simple Hepatectomy group was 4.6065 mmol/L. This finding may be attributable to this study having assessed patients who underwent longer operations as well as more non-simple hepatectomies in comparison with the study by Vibert et al.

Measurement of lactate levels at ICU admission enabled early prediction of postoperative complication incidence, making this measurement a useful monitoring tool. In cases of non-simple hepatectomies, when lactate levels at ICU admission are found to be elevated, we believe there is a need to regularly monitor lactic acid levels, pay close attention to the patient’s subsequent clinical progress, and simultaneously carry out careful systemic management of the patient to reduce lactic acid levels using techniques such as infusion management.

Multiple logistic regression analysis indicated that adjusted ICG, which is an assessment of preoperative liver function, is not an independent factor associated with major complication incidence. We found it highly probable that the preoperative liver function assessment used as the surgical indication criteria at our hospital is appropriate, and that preoperative liver function is not related to the incidence of major postoperative complications.

Among postoperative complications, posthepatectomy liver failure is particularly serious, and its prevention is therefore extremely important. Previous studies have found that posthepatectomy liver failure following hepatectomy occurs at a frequency of between 1.2% and 70%.^18,19^ In the present study, posthepatectomy liver failure of ISGLS grades B and C occurred in 44 patients (38.6%). Of the 44 subjects in the Major Complication Group (C-D III and above), 32 (72.7%) suffered posthepatectomy liver failure. It has been reported that after hepatectomy, immunity and protein synthesis ability decrease, which indicates the possibility of a high risk of postoperative infectious diseases.^1^ The incidence of complications that may be considered part of the “second hit phenomenon,” including infections, has been reported to interfere with resumption of liver function.^20^ In the present study it remained unclear whether there was a causal relationship between postoperative complications and posthepatectomy liver failure, but we believe that preventing postoperative complications or keeping them to a minimum plays an important role in the prevention of further complications.

As far as we were able to determine, no past studies have investigated the relationship between sex and postoperative complications following hepatectomy. Previous studies have reported that women in the ICU with sepsis are at higher risk of ICU mortality^21,22^ but others have also shown that men are at higher risk of ICU mortality,^23^ indicating that there is still no consensus on this matter. The results of the present study, which found that being female is an independent factor associated with major postoperative complications in the Non-Simple Hepatectomy Group, suggest that careful attention should be paid to the postoperative management of female patients.

In addition, the results of exploratory multivariate analysis in this study suggested that in non-simple hepatectomy, ASA PS Class 3, surgical duration, and infusion and blood transfusion volume are possible independent factors in predicting the risk of major postoperative complications.

### Study limitations

The current results should be considered within the context of the study’s limitations. First, because variables that would be related to major postoperative complications were exploratively performed in a multivariate analysis, variables were not restricted. Second, this was a single-center study because the number of extremely long hepatectomies is limited elsewhere (such as in general hospitals) and was also a preliminary study. Third, the number of subjects was small, although we included as many extremely long hepatectomies as possible; however, owing to the rarity of these types of surgeries, the pool of potential cases that we were able to review for inclusion was limited. Fourth, there were missing data for adjusted ICGR_15_ (n=6) and 90-day survival (n=14); hence, these subjects were excluded from the analysis. Although this exclusion was not considered to have greatly affected the statistical analysis, it may have resulted in selection bias.

## Conclusions

In this study, there was a significantly higher number of major postoperative complications arising from non-simple hepatectomies than simple hepatectomies. We examined factors contributing to major postoperative complications in extremely long hepatectomies. In simple hepatectomies, there were no independent factors associated with major postoperative complications. In non-simple hepatectomies, female sex and lactate levels at ICU admission of patients who underwent extremely long hepatectomies lasting 12 or more hours may be independent factors associated with major postoperative complications. Thus, measurement of lactate levels at ICU admission promises to be a useful tool to monitor postoperative clinical progress in patients undergoing non-simple hepatectomies.

## Supplementary Material

Supplementary Material

## Figures and Tables

**Table1:** Comparison of baseline characteristics of all enrolled patients

Variables	Major Complications Group (n=44)	Non-Major Complications Group (n=70)	*p* value
*Patient demographics*			
Age, years	69.0 (63.8–73.5)	66.5 (59.3–73.8)	*0.319*
Sex (male/female), n	24/20	52/18	***0.041***
Height, cm	160.2 (153.0–167.5)	164.9 (157.1–169.2)	*0.138*
Weight, kg	55.9 (50.2–64.0)	62.1 (52.9–69.1)	*0.069*
BMI, kg/m^2^	21.8 (20.6–25.0)	23.2 (21.0–24.7)	*0.185*
DM (yes/no), n	13/31	19/51	*0.832*
Preoperative cholangitis (yes/no), n	3/41	4/66	*1*
Cirrhosis (yes/no), n	5/39	12/58	*0.59*
ASA PS Class 2, n	33	52	*1*
ASA PS Class 3, n	8	3	***0.022***
Child-Pugh score (5/6/7/8), n	27/14/2/1	61/7/1/1	***0.005***
Adjusted ICGR_15_, %	12.9 (9.3–16.9)	9.0 (6.7–12.4)	***0.005***
*Surgical procedure*			
Simple hepatectomy/Non-simple hepatectomy), n	11/33	44/26	***<0.001***
Major hepatectomy (≥2 Couinaud segments), n	30	26	***0.002***
Open hepatectomy/Laparoscopy, n	40/4	36/34	***<0.001***
Redo hepatectomy, n	5	9	*1*
*Intraoperative parameters*			
Surgical duration, h	16.0 (13.9–19.2)	13.9 (12.8–15.4)	***0.001***
Blood loss, mL/kg/h	1.9 (1.4–4.0)	1.1 (0.6–1.8)	***<0.001***
Infusion and blood transfusion volume, mL/kg/h	8.33 (6.65–11.42)	7.25 (5.52–8.75)	***0.008***
Intraoperative use of noradrenaline, n	38	52	*0.159*
Blood inflow occlusion (yes/no), n	38/6	40/30	***0.001***
*Postoperative outcomes*			
Lactate level at ICU admission, mmol/L	4.5 (2.7–6.8)	2.7 (2.1–3.7)	***<0.001***
Posthepatectomy liver failure, n	32	12	***<0.001***
Survival rates			
28-day survival, %	88.6	100	***0.007***
90-day survival, %	78	100	***<0.001***
Hospital stay^a^, days	76 (61–103)	28 (21–48)	***<0.001***

The Major Complications Group was composed of patients with Clavien–Dindo Grade III or more. BMI: Body mass index, DM: Diabetes mellitus, ASA PS: American Society of Anesthesiologists Physical Status, ICGR_15_: Indocyanine green retention rate at 15 min, ICU: Intensive care unit, Redo hepatectomy: Second and subsequent hepatectomy, Blood flow occlusion: Any inflow occlusion technique performed to reduce bleeding during surgery. Posthepatectomy liver failure was defined as International Study Group of Liver Surgery (ISGLS) grade B or C. ^a^ Hospital stay: total in-hospital days including both preoperative and postoperative periods

**Table2:** Adjusted odds ratio for major postoperative complications for all enrolled patients

Parameters	Odds ratio	95% CI	*p* value
Age, years	1.02	0.96–1.08	*0.59*
Sex, female	3.25	0.91–11.6	*0.07*
BMI, kg/m^2^	1.02	0.85–1.23	*0.79*
DM (yes/no), yes	1.03	0.26–4.0	*0.97*
Preoperative cholangitis (yes/no), n	0.89	0.11–7.16	*0.91*
Cirrhosis, yes	0.44	0.07–2.67	*0.37*
ASA PS Class 2, n	3.52	0.58–21.6	*0.17*
ASA PS Class 3, n	11.5	0.99–1.34×10^2^	***0.05***
Adjusted ICGR_15_, %	1.06	0.98–1.16	*0.15*
Open hepatectomy, yes	4.55	0.86–24.1	*0.08*
Major hepatectomy, yes	1.54	0.44–5.41	*0.50*
Redo hepatectomy, yes	0.50	0.07–3.47	*0.49*
Blood inflow occlusion, yes	2.11	0.49–9.17	*0.32*
Surgical duration, h	1.21	1.01–1.46	***0.04***
Blood loss, mL/kg/h	0.98	0.61–1.57	*0.93*
Infusion and blood transfusion volume, mL/kg/h	1.04	0.78–1.38	*0.81*
Intraoperative use of noradrenaline, n	0.69	0.16–3.06	*0.62*
Lactate level at ICU admission, mmol/L	1.16	0.89–1.52	*0.27*

CI: Confidence interval, BMI: Body mass index, DM: Diabetes mellitus, ICGR_15_: Indocyanine green retention rate at 15 min, ASA PS: American Society of Anesthesiologists Physical Status, ICU: Intensive care unit

**Table3:** Comparison of baseline characteristics of enrolled patients undergoing simple hepatectomies

Variables	Major Complications Group (n=11)	Non-Major Complications Group (n=44)	*p* value
*Patient demographics*			
Age, years	71.0 (62.5–73.0)	65.0 (56.0–73.3)	*0.528*
Sex (male/female), n	3/8	9/35	*0.689*
Height, cm	167.0 (162.0–169.8)	166.0 (160.8–170.2)	*0.736*
Weight, kg	62.0 (54.6–77.2)	64.4 (56.8–73.4)	*0.916*
BMI, kg/m^2^	22.1 (21.6–25.6)	24.2 (21.7–26.0)	*0.916*
DM (yes/no), n	5/6	10/34	*0.149*
Preoperative cholangitis (yes/no), n	0/11	2/42	*1*
Cirrhosis (yes/no), n	1/10	10/34	*0.43*
ASA PS Class 2, n	10	33	*0.426*
ASA PS Class 3, n	0	1	*1*
Child-Pugh score (5/6/7/8), n	2/9	4/40	*0.588*
Adjusted ICGR_15_, %	12.5 (10.4–14.6)	9.1 (6.2–11.6)	***0.047***
*Surgical procedures*			
Major hepatectomy (≥2 Couinaud segments), n	3	11	*1*
Open hepatectomy/Laparoscopy, n	8/3	15/29	***0.038***
Redo hepatectomy, n	1	8	*0.667*
*Intraoperative parameters*			
Surgical duration, h	14.9 (14.0–16.3)	13.9 (12.9–14.9)	*0.066*
Blood loss, mL/kg/h	1.7 (0.7–3.0)	0.9 (0.6–1.5)	*0.165*
Infusion and blood transfusion volume, mL/kg/h	6.4 (6.1–8.0)	6.6 (5.4–8.4)	*0.784*
Intraoperative use of noradrenaline, n	8	32	*1*
Blood inflow occlusion (yes/no), n	8/3	23/21	*0.314*
*Postoperative outcomes*			
Lactate level at ICU admission, mmol/L	2.8 (1.7–4.1)	2.6 (2.1–3.7)	*0.983*
Posthepatectomy liver failure, n	5	5	***0.018***
Survival rates			
28-day survival, %	100	100	*1*
90-day survival, %	90	100	*0.213*
Hospital stay^a^, days	74 (52–98)	25 (21–39)	***0.001***

The Major Complications Group was composed of patients with Clavien–Dindo Grade III or more. BMI: Body mass index, DM: Diabetes mellitus, ASA PS: American Society of Anesthesiologists Physical Status, ICGR_15_: Indocyanine green retention rate at 15 min, ICU: Intensive care unit, Redo hepatectomy: Second and subsequent hepatectomy, Blood inflow occlusion: Any inflow occlusion technique performed to reduce bleeding during surgery. Posthepatectomy liver failure was defined as International Study Group of Liver Surgery (ISGLS) grade B or C. ^a^ Hospital stay: total in-hospital days including both preoperative and postoperative periods

**Table4:** Adjusted odds ratio for major postoperative complications in patients undergoing simple hepatectomies

Parameters	Odds ratio	95% CI	*p* value
Age, years	1.29	0.94–1.78	*0.12*
Sex, female	4680	0.37–6.0×10^7^	*0.08*
BMI, kg/m^2^	0.57	0.27–1.22	*0.15*
DM (yes/no), yes	1530	0.41–5.69×10^6^	*0.08*
Cirrhosis, yes	0.01	0.00–2.76	*0.10*
Adjusted ICGR_15_, %	1.00	0.59–1.66	*0.97*
Open hepatectomy, yes	28500	0.36–2.24×10^9^	*0.07*
Major hepatectomy	35.2	0.14–8.67×10^3^	*0.2*
Redo hepatectomy, yes	0.07	0.00–13.3	*0.32*
Blood inflow occlusion, yes	56.1	0.16–1.93×10^4^	*0.18*
Surgical duration, h	2.45	0.71–8.40	*0.16*
Blood loss, mL/kg/h	1.85	0.20–16.8	*0.58*
Infusion and blood transfusion volume, mL/kg/h	0.17	0.02–1.37	*0.09*
Intraoperative use of noradrenaline, yes	0.20	0.00–17.8	*0.48*
Lactate level at ICU admission, mmol/L	0.48	0.15–1.53	*0.21*

Preoperative cholangitis and American Society of Anesthesiologists Physical Status were excluded from the analysis because of multicollinearity CI: Confidence interval, BMI: Body mass index, DM: Diabetes mellitus, ICGR_15_: Indocyanine green retention rate at 15 min, ICU: Intensive care unit

**Table5:** Comparison of baseline characteristics of enrolled patients undergoing non-simple hepatectomies

Variables	Major Complications Group (n=33)	Non-Major Complications Group (n=26)	*p* value
*Patient demographics*			
Age, years	69.0 (64.0–75.0)	67.0 (62.0–75.0)	*0.891*
Sex (male/female), n	16/17	17/9	*0.291*
Height, cm	158.3 (151.0–165.0)	161.5 (153.3–166)	*0.445*
Weight, kg	54.3 (47.9–62.8)	57.7 (48.3–63.7)	*0.593*
BMI, kg/m^2^	21.6 (19.8–23.1)	21.9 (20.2–23.8)	*0.855*
DM (yes/no), n	8/25	9/17	*0.403*
Preoperative cholangitis (yes/no), n	3/30	2/24	*1*
Cirrhosis (yes/no), n	4/29	2/24	*0.685*
ASA PS Class 2	23	19	*1*
ASA PS Class 3	8	2	*0.161*
Child-Pugh score (5/6/7/8), n	18/12/2/1	21/3/1/1	*0.093*
Adjusted ICGR_15_, %	11.6 (9.5–19.0)	11.1 (7.2–13.8)	*0.147*
*Surgical procedure*			
Major hepatectomy (≥2 Couinaud segments), n	27	15	***0.05***
Open hepatectomy/Laparoscopy, n	32/1	21/5	*0.078*
Redo hepatectomy, n	4	1	*0.372*
*Intraoperative parameters*			
Surgical duration, h	16.5 (13.9–19.3)	14.1 (13.0–16.7)	***0.036***
Blood loss, mL/kg/h	2.2 (1.4–4.1)	1.4 (0.7–2.4)	***0.013***
Infusion and blood transfusion volume, mL/kg/h	9.2 (7.8–12.2)	8.4 (6.2–9.3)	*0.072*
Intraoperative use of noradrenaline, n	30	20	*0.164*
Blood inflow occlusion (yes/no), n	30/3	17/9	***0.023***
*Postoperative outcomes*			
Lactate level at ICU admission, mmol/L	5.2 (3.4–7.2)	2.9 (2.4–3.7)	***0.002***
Posthepatectomy liver failure, n	27	7	***0.005***
Survival rates			
28-day survival, %	84.8	100	***0.061***
90-day survival, %	74.2	100	***0.015***
Hospital stay^a^, days	77 (61–114)	44 (28–74)	***<0.001***

The Major Complications Group was composed of patients with Clavien–Dindo Grade III or more. BMI: Body mass index, DM: Diabetes mellitus, ASA PS: American Society of Anesthesiologists Physical Status, ICGR_15_: Indocyanine green retention rate at 15 min, ICU: Intensive care unit, Redo hepatectomy: Second and subsequent hepatectomy, Blood inflow occlusion: Any inflow occlusion technique performed to reduce bleeding during surgery. Posthepatectomy liver failure was defined as International Study Group of Liver Surgery (ISGLS) grade B or C. ^a^ Hospital stay: total in-hospital days including both preoperative and postoperative periods

**Table6:** Adjusted odds ratio for major postoperative complications in patients undergoing non-simple hepatectomies

Parameters	Odds ratio	95% CI	*p* value
Age, years	1.05	0.92–1.20	*0.44*
Sex, female	13.4	1.00–1.81×10^2^	***0.04***
BMI, kg/m^2^	0.97	0.65–1.43	*0.88*
DM, yes	0.57	0.06–5.00	*0.61*
Preoperative cholangitis (yes/no), yes	0.82	0.04–13.7	*0.89*
Cirrhosis, yes	1.70	0.04–60.1	*0.76*
ASA PS Class 2, n	10.8	0.27–4.27×10^2^	*0.20*
ASA PS Class 3, n	80.8	0.80–8.08×10^3^	*0.06*
Adjusted ICGR_15_, %	1.07	0.93–1.22	*0.31*
Open hepatectomy, yes	0.68	0.007–67.7	*0.87*
Major hepatectomy, yes	3.42	0.30–38.1	*0.31*
Redo hepatectomy, yes	0.28	0.006–13.3	*0.52*
Blood inflow occlusion, yes	2.83	0.10–77.7	*0.53*
Surgical duration, h	1.30	0.94–1.80	*0.10*
Blood loss, mL/kg/h	0.62	0.28–1.34	*0.22*
Infusion and blood transfusion volume, mL/kg/h	1.46	0.88–2.42	*0.13*
Intraoperative use of noradrenaline, yes	0.32	0.02–4.79	*0.41*
Lactate level at ICU admission, mmol/L	1.60	0.99–2.59	***0.05***

CI: Confidence interval, BMI: Body mass index, DM: Diabetes mellitus, ICGR_15_: Indocyanine green retention rate at 15 min, ASA PS: American Society of Anesthesiologists Physical Status, ICU: intensive care unit

## Supplementary data

Supplementary data are available on the J-STAGE.
