# Supplementary material for: Retrospective study of predictive factors for postoperative complications of hepatectomies lasting 12 or more hours
Source: Fujita Med J. 2020 Feb 11;6(3):59–66. doi: 10.20407/fmj.2019-003 (PMC8749506; doi:10.20407/fmj.2019-003)
Supplement: Supplementary file 1 — Supplementary Material [file fmj-6-059_s1.pdf]

Supplementary Table 1. Postoperative complications that developed in each Clavien–Dindo grade

Complication	Clavien–Dindo classification							Total, n
	I (n=8)	II (n=29)	IIIa (n=23)	IIIb (n=5)	IVa (n=5)	IVb (n=8)	V (n=3)	
Liver failure		12	15	3	4	7	3	44
Bile leakage	1		5	2				8
Cholangitis		2	1			1		4
Pancreatic leak			1					1
Ascites		4						4
Pleural effusion		2	8		1		1	12
Pneumonia			1	1		1		3
Cerebral infarction						1		1
Intestinal ischemia							1	1
Postoperative bleeding				1	2	2		5
Thrombosis		1	1		2			4
Abscess		1	5					6
Wound infection		1						1
Deviation of wound				1				1
Suture failure						1		1
Ileus			1					1
Others	7	11	4	1	1	5	3	32

Others: fever, pneumothorax, acute respiratory distress syndrome, acute kidney injury, pseudoaneurysm, sepsis, peritonitis, enteritis

Supplementary Table 2. Diseases and surgical procedures

Diseases requiring surgery	Major Complications Group (n=44)	Non-Major Complications Group (n=70)
Hepatocellular carcinoma	12 (5)	31 (2)
Metastatic liver tumors	12 (6)	26 (2)
Perihilar cholangiocarcinoma	10 (8)	4 (4)
Intrahepatic cholangiocarcinoma	6 (6)	6 (4)
Distal cholangiocarcinoma	0 (0)	1 (1)
Other liver tumors	3 (2)	2 (1)
Gallbladder cancer	1 (1)	0 (0)
Total	44 (29)	70 (14)

Numbers in parentheses denote those of cases where concomitant vascular and/or bile duct resection with reconstruction was carried out
